# Systematic use of transvaginal hydrolaparoscopy as a minimally invasive procedure in the exploration of the infertile patient: results and reflections

**DOI:** 10.52054/FVVO.13.2.014

**Published:** 2021-06-28

**Authors:** S Gordts, Sy Gordts, P Puttemans, I Segaert 1,2, M Valkenburg, R Campo

**Affiliations:** Life Expert Centre, Schipvaartstraat 4, 3000 Leuven, Belgium; Heilig Hart Hospital, Naamsestraat 104, 3000 Leuven, Belgium.

**Keywords:** Transvaginal hydrolaparoscopy, diagnosis, ovarian drilling, endometriosis

## Abstract

**Background:**

The aim of this study was to evaluate the added value of transvaginal hydrolaparoscopy (THL) in the investigation of the infertile patient.

**Methods:**

A retrospective cohort study, based on records from 01/09/2006 to 30/12/2019 was undertaken in a tertiary care infertility centre. THL was performed in 2288 patients. These were patients who were referred for endoscopic exploration of the female pelvis as part of their infertility investigation. In 374 patients with clomiphene- resistant polycystic ovary syndrome (PCOS), ovarian capsule drilling was also performed. The outcome objectives of this study included the evaluation of the added diagnostic value of THL as well as the feasibility and safety of the visual inspection of the female pelvis using this technique.

**Results:**

Of the 2288 procedures failed access to the pouch of Douglas occurred in in 23 patients (1%). The complication rate was 0.74%, due to bowel perforations (n= 13) and bleeding (n= 4) requiring laparoscopy. All bowel perforations were treated conservatively, with 6 days of antibiotics, and no further complications occurred. Findings were normal in 49.8% of patients. Endometriosis was diagnosed in 366 patients (15.9%); adhesions were present in 144 patients.

**Conclusions:**

THL is a minimally invasive procedure, with a low complication and failure rate, providing an accurate visual exploration of the female pelvis in a one-day hospital setting. When indicated, minimally invasive surgery is possible in the early stages of endometriosis and for ovarian capsule drilling in patients with clomiphene- resistant PCOS.

## Introduction

Transvaginal hydrolaparoscopy (THL) was introduced as a minimally invasive procedure for the exploration of the female pelvis in patients trying to conceive for at least one year and in absence of obvious pelvic pathology as confirmed by a normal vaginal examination and normal vaginal ultrasound (Gordts et al., 1998, Campo et al., 2002, Watrelot et al., 2003, Tanos et al., 2005). In the exploration of the infertile patient and with the increasing accuracy of ultrasound (US), the added value of a laparoscopy as a pure diagnostic tool has been questioned and is frequently postponed or not performed anymore. The invasiveness of the diagnostic laparoscopy is balanced against an easy referral to advanced reproductive technology (ART) programs. Although US is a valuable first line screening method to exclude severe pelvic or uterine pathology and to exclude contra-indications for the THL, direct visualisation of the pelvis with endoscopy remains the gold standard to exclude minor endometriotic lesions and the presence of tubo-ovarian adhesions. Indeed, tubal infertility and endometriosis are well known underlying causes in patients with fertility problems. In addition to patency, tubal function relies upon the functions of the fimbriae and the ciliated cells of the tubal mucosa and is impaired by the presence of filmy, peri-tubal and peri-ovarian adhesions. Therefore, the pelvis should be explored to detect endometriosis and tubal function in the fertility work-up to avoid overestimating unexplained infertility. In THL a watery solution, preferably Hartmann solution, is used for distension increasing the accuracy of detecting early endometriosis and identifying filmy adhesions (Gordts et al., 2000). The procedure can be performed in an outpatient setting, with the patient under local anesthesia or sedation; it was shown to be a safe procedure with a learning curve of 50 procedures (Gordts et al., 2001, Verhoeven et al., 2004). In this paper, we describe our experiences over more than a decade with the systematic use of THL as a first line diagnostic procedure for exploring the female pelvis in a consecutive cohort of patients seeking fertility treatment.

## Materials and methods

**Figure 1:**
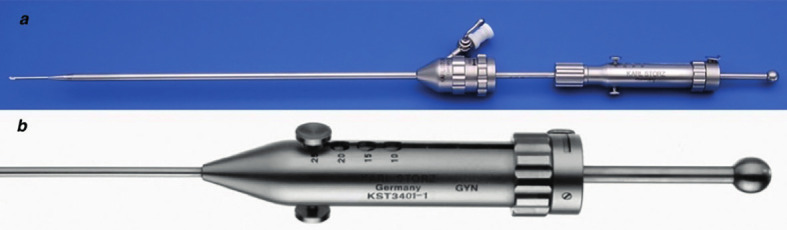
— Reusable spring-loaded needle set. a. Assembled set consisting out of the needle, dilating device and outer trocar b. Detail of spring-loaded needle: length of needle point can be preset between 10 and 25mm.

This retrospective study included 2288 patients that visited our fertility clinic for primary (80.7%) or secondary (19.3%) infertility and underwent THL as part of the fertility work-up between September 2006 and December 2019. In the absence of an absolute indication for in vitro fertilisation (IVF) such as severe male infertility, or in the case of pre- implantation genetic testing, all patients seeking help for their fertility problems were referred for a one stop fertility exploration including a diagnostic hysteroscopy and transvaginal hydrolaparoscopy. Prior to the THL referral, all patients provided a detailed history and underwent a vaginal examination and ultrasound to confirm the absence of obvious pelvic pathology. As a patency test was performed during the THL, we omitted hysterosalpingography as a routine examination in our fertility exploration. Contra-indications for THL were the presence of recto-vaginal pathology, a fixed retroverted uterus, and endometriotic cysts larger than 2 cm in diameter. All THL procedures were performed in a single facility by three gynecologists with extensive experience in the THL technique. We have described the THL technique previously (Gordts et al., 1998, Campo et al., 2002). In summary, the pelvis is accessed by a simple needle puncture of the pouch of Douglas. In contrast to fertiloscopy (Watrelot et al., 1999), where access is gained with a single-use instrument, in THL, the pelvis is accessed with a reusable, spring-loaded needle with an adjustable length (1.0 cm to 2.5 cm), which proved to be advantageous when dealing with obese patients. The assembled pelvic access system comprised the spring-loaded needle, a dilating device, and an outer trocar (3.9 mm diameter; Storz, Germany) (Figure 1).

The advantage of the spring-loaded needle is the speed once the needle is shut, which avoids tenting of the peritoneum and minimises the risk of access failure. All procedures were performed in a one-day hospital setting. Patients were placed under conscious sedation, in a gynecological position, without Trendelenburg. Although THL can be performed under local anesthesia, in our experience when the procedure is performed in an operating room, patients tend to be more nervous and sedation is preferable. Moreover, when it was necessary to perform some minor interventions, this can easily be done avoiding the need to switch from local anesthesia to conscious sedation. In the same session, prior to the THL, a vagino-cervico- hysteroscopy was routinely performed in all patients that had not received one previously. When the fallopian tubes were patent, the hysteroscopy allowed overflow of fluid into the pouch of Douglas. Patency was tested by introducing a Foley catheter no. 8 into the uterine cavity. The complete procedure of diagnostic hysteroscopy, THL and patency testing takes between 20-30 minutes and provides an accurate picture of implantation, tubal patency and transport while excluding pelvic pathology like peri- tubal adhesions and peritoneal or minimal ovarian endometriosis. As the procedure is minimally invasive, patients can leave after one hour.

**Figure 2:**
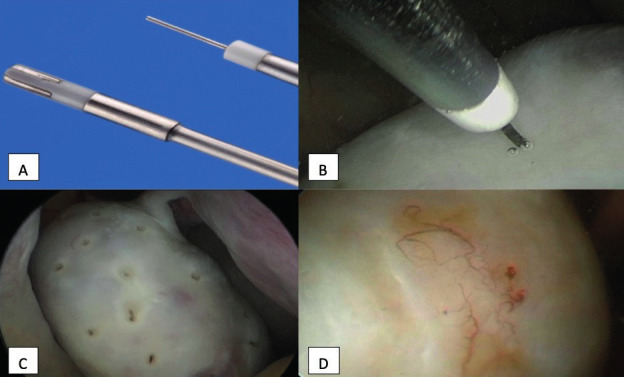
— Ovarian capsule drilling through THL. A: 5Fr. bipolar needle and bipolar probe; B: Drilling of ovarian capsule by bipolar needle placed perpendicular to ovarian surface; C: Overview of drilled ovarian surface; D: Second look after a previous drilling; presence of only small angiogenesis and no adhesions.

For ovarian drilling, the inclusion criteria were: primary or secondary infertility; a PCOS diagnosis, clomiphene-resistance; and no contraindication for a THL procedure. The drilling technique has been described previously (Gordts et al., 2009). Briefly, it was performed under sedation and a fine bipolar needle was used (Figure 2) to make 5-10 small holes in the ovarian capsule.

All patients provided informed consent before the procedure. In addition, patients were informed that, in case of complications, the operation could be converted to a standard laparoscopy. All patients agreed that their data could be used anonymously for research. This retrospective study involved the collection of existing data recorded for procedures routinely performed in exploring the pelvis of infertile patients; therefore, institutional review board approval was not mandatory.

## Results

### 

We identified 2288 patients with a mean age of 31.25 ± 3.8 years (SD). The mean duration of infertility was 23.6 months (5^th^-95^th^ percentile: 11-48). Among these 2288 patients, 1914 underwent THL for diagnostic purposes only, and 374 patients were referred for ovarian drilling. Inspection revealed the presence of endometriosis in 366 patients (15.9%), adhesions in 144 patients due to a previous infection, and hydrosalpinx in 26 patients. Patency testing showed abnormal patency in one or both tubes in 139 patients. Findings were normal in 49.8% of patients (Table I).

**Table I:** Findings at THL in the period 01/09/2006- 30/12/2019.

Number procedures		2288
Normal findings (%)		1128 (49.3%)
Endometriosis		366 (15.9%)
Tubal pathology		334 (14.5%)
	Adhesions (infectious)	144
	Hydrosalpinx	26
	Fimbrial abnormality	51
	Failed patency	139
Ovarian drilling		387
Failed access		23 (1%)
Complications at access		17 (0.74%)
	Bowel perforation	13
	Bleeding	4

Failure to access the pouch of Douglas occurred in 23 patients (1%). The complication rate was 0.74%; complications included perforation of the bowel (n= 13) and bleeding that required laparoscopy (n= 4).

### Tubal pathology

**Figure 3:**
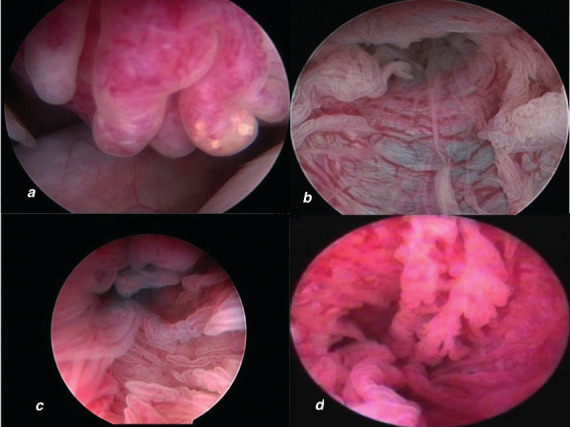
— Fimbrioscopy and salpingoscopy at THL. a. abnormal plumb aspect of fimbriae; b. dilated folds due to hypoplastic tubal muscular wall; c. salpingoscopy showing normal tubal mucosa with major and minor folds; d. abnormal tubal mucosa at salpingoscopy.

Patency was tested by inserting a Foley catheter (number 8) into the uterine cavity at the beginning of the procedure and flushing with diluted methylene blue. In 139 patients, patency could not be visualised on one (n= 97) or both sides (n=42). We identified 26 hydrosalpinges not diagnosed at US. Other abnormalities in the fimbriae or distal ampulla were detected in 51 patients and 84.3% of these abnormalities were bilateral. In these patients, the fimbriae appeared rather fibrotic and plump (Figure 3). We found no correlation between occlusions and the presence of chlamydia antibodies.

Post inflammatory peri-tubal and/or peri-ovarian adhesions were diagnosed in 144 patients (6.3%). Patients with infectious tubal pathology were given preventive antibiotics. Data were not systematically recorded regarding the presence or absence of cysts of Morgagni.

### Endometriosis

**Figure 4:**
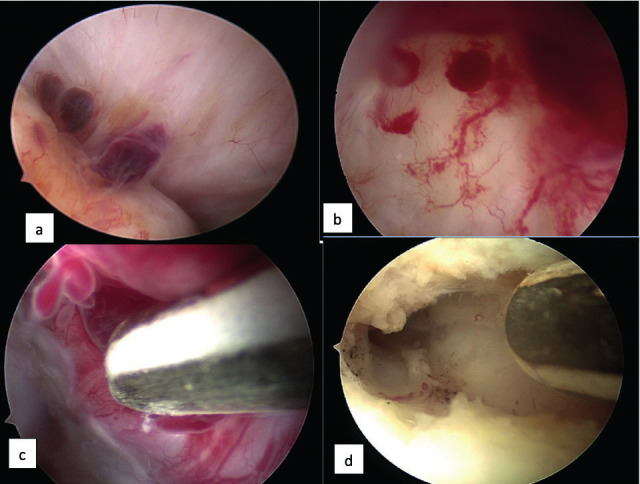
— THL and endometriosis. a. Endometriotic lesions and adhesions fixing ovary in fossa; b. Inside view of endometrioma showing the inflammatory reaction with neo-angiogenesis and presence of endometrial like tissue; c. 5Fr bipolar probe for coagulation of inner wall of small endometrioma; d. Final result after bipolar coagulation; remark white surface and absence of carbonization.

Endometriotic lesions were diagnosed in 15.9 % (n=366) of patients. Positioning the scope during THL provided direct access to the tubo- ovarian structures and the fossa ovarica, without extra manipulation. This contrasts with standard laparoscopy, where the ovary must be grasped and lifted to inspect the fossa ovarica; this manipulation runs the risk of rupturing adhesions and causing bleeding. In THL, the close contact with organs and the use of a watery distension medium enabled the detection of early endometriosis. Even when the lesions were small, the presence of inflammation and neo-angiogenesis reflected disease activity (Figure 4).

The incidence was higher on the left side (n=237) than on the right side (n=169). Lesions appeared as brown or red vesicles on the ovarian or peritoneal surface, covered with filmy adhesions, and signs of neoangiogenesis. When the ovary was fixed with filmy or more fibrous adhesions in the fossa, endometrial-like tissue was identified at the moment of dissection. Small endometriomas, with diameters between 0.5 and 2 cm, were present on the right side in 31 patients, on the left side in 48 patients, and bilateral in 10 patients. After opening and draining these small endometriomas, an ovarioscopy confirmed the presence of neoangiogenesis, blebs of endometrial-like tissue, and in some cases, a fibrotic wall. All cysts caused an invagination of the ovarian cortex; in addition, adhesions covered the site of invagination or fixed the ovary to the pelvis in the fossa ovarica. With a 5-Fr bipolar coagulation probe (K. Storz, Germany) (Figure 4) the cystic wall was coagulated, which resulted in a white surface without carbonisation and with minimal trauma. This treatment can be discussed, but in the absence of specific biomarkers, it will remain unclear whether these lesions will fade over time or evolve to more severe stages of endometriosis. Due to the presence of multiple adhesions or extensive endometriotic lesions, laparoscopy was indicated in 19 patients. Among these, 9 patients refused laparoscopy. In the other 10 patients, laparoscopy confirmed THL findings of several peri-ovarian and peri-tubal adhesions, which were too extensive for THL treatment. The detection of these lesions at THL demonstrates the added value of the endoscopic exploration of the pelvis in patients with normal clinical findings and normal US.

### Ovarian drilling

THL was performed for ovarian drilling in 387 patients. Inclusion criteria were patients with primary (80%) or secondary (20%) infertility with PCOS diagnosis, clomiphene resistant and no contra-indication for a THL procedure.

Results in terms of pregnancy rates are comparable with the results obtained after laparoscopy with less risk of postoperative adhesion formation (Giampaolino P et al., 2016, Giampolino P et al., 2017, Elkelani OA et al., 2002). Conversion to laparoscopy was necessary in two patients: one due to access failure and one due to the presence of adhesions and endometriosis in the pouch of Douglas, which resulted in an incomplete THL. In two other patients, the drilling was incomplete, and a laparoscopy was suggested, but these patients preferred to go directly to IVF treatment.

In our series of 387 patients, five (1.3%) developed severe abdominal pain a few hours after the procedure. When we were faced initially with this problem a laparoscopy was performed in the first two patients to assess the potential causes of pain. No clear cause was identified and conservative management is now advised when patients complain of pain. This involves antiphlogistic medication and an overnight hospital stay if needed.

### Failures and complications

Failures and complications are divided into those related to the transvaginal access and those related to the operative procedure itself.

Related to the transvaginal access, we were not able to access the pelvis in 23 patients.

Routinely, when we could not access the pouch of Douglas after three attempts, we stopped the procedure to avoid potential complications. Previously, obese patients were at higher risk of access failure than non-obese patients, due to an increased incidence of peritoneal tenting. However, with the ability to pre-set the length of the spring- loaded needle, this elevated risk of failed access was reduces. In case of access failure, laparoscopy was performed in 8 patients (n=2 on the same day, n=6 at a later time). In three patients, access failure was due to the problem of peritoneal tenting. In one patient the pelvis was completely normal at laparoscopy and no reason for access failure could be identified. Among the other 4 patients, access failure could be explained by the presence of adhesions in the pouch of Douglas (n=2), a fixed ovary (n=1), and the presence of endometriosis in the pouch of Douglas (n=1). The remaining 15 patients refused to proceed to laparoscopy.

In 13 patients (0.56%), the procedure was complicated by a perforation of the rectum. In contrast to intestinal perforations at a standard laparoscopy where the risk of a late recognition is real, here the perforation is directly visualised. In THL it is particularly important to keep the scope stationary and avoid excessive movement to reduce the risk of enlarging the injury. Under visual control the endoscope is gently removed and the procedure is stopped. Per-operatively antibiotics are administered intravenously and patients were further treated with antibiotics for 6 days and asked to record their temperature and to contact our unit in case of any abnormality. None of the patients displayed further complications or signs of infection. In 5 patients, we performed a standard laparoscopy at a later stage. One patient had a frozen pelvis, and two patients had pathology of the sacro-uterine ligaments, due to the presence of fibrosis and a small nodulus. In the remaining patients, no specific reason could be found explaining the perforation.

In the diagnostic procedures the access was complicated by a bleeding in 4 patients requiring conversion to standard laparoscopy. In 2 patients bleeding was caused by an inadvertent puncture of the posterior site of the uterus, in one patient due to puncture of the parametrium and in one patient due to puncture of the pararectal fat. These complications were easily resolved at standard laparoscopy and patients were discharged the same day.

In the operative procedures bleeding occurred in 9 patients caused by the intervention itself. In 6 patients bleeding was noted at the moment of drilling of the ovarian capsule and in 3 patients by treating the endometriotic lesions. Using the 5 Fr. bipolar coagulation probe (K. Storz, Germany) the bleeding was easily stopped in all patients undergoing the ovarian drilling and in 2 of the patients treated for endometriosis. In one patient a standard laparoscopy was undertaken due to venous bleeding at the hilus of the ovary and hemostasis was obtained. Although no prospective recording was done on post-operative complications, all patients were reviewed at 4 weeks. A case note review was also undertaken which did not indicate any further complications. No long terms complications or complaints of persistent pain were noted in the cohort of bowel complications.

## Discussion

THL is a minimally invasive procedure for exploring the female pelvis. The use of a watery distension medium and close inspection guarantees high accuracy in the detection of minimal lesions. We demonstrated that, in cases of minimal endometriosis, THL detected more adhesions than could be detected with standard laparoscopy (Brosens et al., 2001). Compared to standard laparoscopy, THL sensitivity was 70-88%, with 100% specificity (Watrelot et al., 2003, Darai et al., 2000, Dechaud et al., 2001). In a recent study (Coenders-Tros et al., 2016), THL sensitivity was 100%, but specificity was only 22%, due to the fact that laparoscopy was performed only in a few women that showed no abnormalities with THL. In this study, we only selected patients for THL when they had normal findings at clinical examinations and vaginal US. Consequently, THL confirmed normal findings in 49.8% of patients, comparable to findings in other studies (Watrelot, 2007). All these women were spared a standard laparoscopy with general anesthesia; thus, THL provided an advantage in its ability to allow direct visualisation of the female pelvis. Evaluation of tubal pathology still remains important in the exploration of the infertile patient. Hysterosalpingography (HSG) is considered a first line investigation for testing tubal patency. Compared to THL, HSG has a lower sensitivity of 65% and a higher specificity of 83% (Swart et al. 1995). A study evaluating the findings of THL in a cohort of 51 patients with abnormal findings at HSG (Yang Rui et al., 2011) found normal findings in 26 patients (50.9%) at THL. Of the remaining 23 patients, 6 were treated using THL and 4 of them became pregnant spontaneously or with intra uterine insemination (IUI). Shibahara et al. (2001) found no discrepancy in tubal patency testing in patients with and without C. trachomatis infection but revealed the presence of peri-tubal adhesions in 58.3% in patients with past C trachomatis infection and in 18.2% in patients without C. Trachomatis infection. False positive results at HSG were found in 60 % of the patients showing normal findings at laparoscopy (Tanahatoe et al., 2008). These false positive results at HSG present the physicians an “IVF or not “dilemma. In case of a normal and well performed HSG, one must be aware that possible pathology is missed like endometriosis and peri-tubal adhesions; in case of abnormal HSG an endoscopic exploration of the pelvis is strongly advised. Limitations encountered with hysterosalpingo contrast sonography (HyCoSy) include difficulties visualising the entire Fallopian tube, to see and interpret the spilling of the contrast medium and the requirement of an experienced sonographer (Exacoustos et al., 2009). As such results were inconclusive in 6.1- 21.9% of women (Dreyer et al., 2014; Ludwin et al., 2017; Emanuel and Exalto., 2012).

In this consecutive series of patients, our access failure rate was only 1%, which was lower than previous reported rates of approximately 3%. This lower failure rate could be explained by our experience, the selection of patients, and the use of a spring-loaded needle. In a study by Tros et al. (2020), the access failure rate was 6.8%; however, they included procedures performed during training, and the procedures were performed by 16 gynecologists. It is known that complication rates are the highest in the first 50 procedures, and a steady decrease in the rate occurs as more experience is gained (Gordts et al., 2001, Coenders-Tros et al., 2016, Chapron et al., 1999). Most gynecologists are not familiar anymore with the transvaginal access and it requires training. In a multinational retrospective study, we demonstrated that after 50 procedures the rate of bowel perforations decreased from 1.3% to 0.3%. (Gordts et al., 2001). This impact of training or experience was also described in the study of Coenders-Tros et al. (2016) with a decrease in complication rate from 5% (first 50 procedures) to 2% (between 50-100) and 0.7% (>100 procedures). Only by introducing the transvaginal access in gynecological training programs can a more widespread use of the technique be undertaken, which could easily be carried out in any adequately equipped outpatient facility.

Our complication rate was 0.74%. In cases of bowel perforation (0.56%), none of the patients required conversion to laparoscopy or laparotomy; instead, they received conservative treatment with antibiotics. We found that, when no extra manipulation was performed at the moment of perforation, which was directly visible, the hole in the rectum had a maximum diameter of 4 mm, and the elasticity of the muscular rectal layer caused the hole to shrink, once the instrument was removed. This observation was consistent with findings in previous studies (Gordts et al., 2001, Tros et al., 2020). Consequently, bowel perforations were considered a minor complication of the THL technique, and they were not comparable to the high-risk intestinal perforation, which can occur in a standard laparoscopy. In standard laparoscopy, most severe complications are caused by missing the identification of the perforation with pelvic- peritonitis occurring after a few days (Chapron et al., 1999). Moreover, these perforations at THL frequently occur when adhesions are present in the pouch of Douglas; so that most of the lesions are localised extra-peritoneally. The concomitant use of ultrasound guidance when gaining access to the pouch of Douglas was reported to reduce the risk of bowel perforations, certainly in the initial learning period (Sobek et al., 2008, Ma et al., 2012). The cumulative results and experiences of several centres performing the THL procedure highlighting low minor complication rates and low access failure rates are summarised in Table II.

**Table II:** Earlier and more recent publications confirming the low failure and complication rates.

	Number	Failure access	Complications	Normal	Endo/adh
Van Tetering EA. et al. 2007	272	4%	2%	78%	8%
Tanos V. et al. 2005	78	3.8%	1.2%	38%	9-20%
Rui Yang et al. 2011	51	3.9%	0	53.1%	11.7%
Watrelot A. et al. 1999	160	3.8%	0.6%	37.5%	13.1/36.2%
Watrelot A. et al. 2003	92	5.4%	3.2%	30.8%	
Yi-Xin et al. 2014	510	2.9%	0.98%	15.9%	16.5%/44%
Coenders-Tros et al. 2016	1103	6.8%	2.6%	70.9%	6.2%/4.1%
Vanspranghels R. et al. 2020	123	3.2%	0	78.2%	8%/8%
Kissler S. et al. 2011	239	0.8%	2.1%	67.5%	32.5%
Verhoeven H. et al. 2004	1000	1.1%	2.3%	73.6%	25%
Campo R. et al. 2001	349	5.5%	0.9%	55.8%	18%/11.4%
Gordts S. et al. 2008 (review)	2843	6%	0.74%		

Nevertheless, there still is a resistance to the uptake of THL which already existed in the past. Furthermore, Diamond (1978) published a series of 4000 culdoscopies and concluded that the use of diagnostic culdoscopy as an outpatient procedure provided better access for the diagnosis and treatment of infertility, especially when the pathology is not extreme enough to warrant a laparoscopy. He advised that the technique should return to the gynecological training programs and he concluded:

“True culdoscopy requires laboriously won special skills, but its advantage to patient and physician are well worth the trouble. Once mastered culdoscopy equips the gynecologic endoscopist with a rapid and minimally traumatic outpatient option that supplies rich information not only in the initial diagnosis of infertility but also in circumstances where laparoscopy might be inappropriate”.

Although THL differs from culdoscopy by the position of the patient and the use of a fluid distension medium instead of CO2, the basic principles of the procedure are the same. The primary challenge of changing established attitudes remains always a burden and is difficult even in the presence of grade A evidence as seen in daily practice where hysteroscopy still is not replacing dilation and curettage (D&C) despite its diagnostic and therapeutic superiority. Secondly the training programs in gynaecology concentrates upon the treatment of gynaecological pathologies with standard laparoscopy and does not incorporate training in reproductive surgery as a standards. A third reason is the fear of rectal perforation, which in the initial period is understandable, but with the available data today may not be a limiting factor anymore (Table II). A fourth reason is that most of the gynaecologists involved in reproductive medicine are reducing their therapeutic compendium to hormonal treatments, IUI and IVF; this is reflected in the several stand-alone centers for ART. This situation feeds the lack of interest in an accurate diagnosis and in the importance of a spontaneous conception. If the first goal of infertility treatments should be to create the highest possibility for a spontaneous conception, the transvaginal approach should become part of the training programs in reproductive surgery in university hospitals and in the relevant societies.

Although THL was developed as a first line diagnostic tool, it became clear that, during the THL, minimal operative procedures could be performed. The range of possible procedures is limited by the absence of a panoramic view and the close proximity to organs. Accordingly, we found that it was possible to perform small endometriotic lesion excisions, limited adhesiolysis, and ovarian drilling.

The systematic omission of endoscopic visualisation of the pelvis in the exploration of the infertile patient results in undiagnosed lesions of endometriosis, tubal pathology and adhesions and an increased incidence of the so called” unexplained infertility”. The cohort of patients referred to IVF in our centre with the diagnosis of unexplained infertility is 5.83% versus a mean of 21.63% in other Belgian centers, as documented in the Belrap report of 2017 (Belrap, 2017). Recent studies have revealed a high incidence of untreated endometriosis in patients with “unexplained” infertility, and those missed lesions had a negative impact on pregnancy rates (Evans-Hoecker et al., 2016, Almquist et al., 2017). THL is a valuable option for these patients (Tros et al. 2019, Balsac et al., 2014). Pantou et al. (2019) reported the presence of endometriosis in 57.94% and 23.3% in a cohort of 107 patients with unexplained infertility referred for laparoscopy after 3 failed IVF attempts. Correcting these pathologies at laparoscopy resulted in a spontaneous cumulative pregnancy rate of 50-60 %. In a smaller study including 45 patients with unexplained infertility and failed IVF, Yu et al., (2019) reported the presence of endometriosis, tubal pathology and adhesions in 57.7 %, 31.1% and 33.3 % of the patients respectively. In the study by Yoshiaki et al. (2006) the diagnosis of unexplained infertility could only be confirmed in 39.8% of patients when a laparoscopy was performed revealing presence of endometriosis (50.7%), tubal pathology (6.5%) and adhesions (2.8%) in the other patients.

In transvaginal access, the axis of the endoscope is parallel to the longitudinal tubo-ovarian axis. Without supplementary manipulation, easy access is gained to the tubal ampullary part with the possibility of salpingoscopy in 50% of the attempted tubes dependent on the mobility of the tubes (Gordts et al., 1998, Suzuki et el., 2005). In close proximity to the fimbrial end, a detailed inspection of the fimbriae can reveal subtle pathology (Figure 3). This pathology could potentially cause a disturbance in the ovum pick-up mechanism at the moment of ovulation (Gordts et al., 1998). Indeed, the fine-tuned process of ovum pick-up and transport can be impaired by the presence of filmy intra-fimbrial adhesions, peri-tubal adhesions, and cysts of Morgagni. Even in presence of normal patency, aspects of fimbrial and tubal mucosa are important in the prognosis for spontaneous conception. Spontaneous pregnancies have been reported after these subtle lesions are surgically corrected (Guan and Watrelot, 2019, Rasheed and Abdelmonem, 2011). In cases of hydrosalpinx, THL offers the possibility of opening the tubes and exploring the tubal lumen and mucosa. This information can facilitate a decision between performing a salpingostomy, which could provide the possibility of a spontaneous conception, or performing a salpingectomy and referring the patient for IVF (Heylen et al., 1995, Audebert et al., 2014).

## Conclusion

Currently, a standard laparoscopy is frequently omitted in the exploration of the female pelvis due to its relative invasiveness for a simple diagnostic procedure. THL offers the potential of a minimally invasive procedure for this exploration, with the patient under local anesthesia or sedation, avoiding the inconveniences of a standard laparoscopy. Training is mandatory to become familiar with the tactile sensation to access the pouch of Douglas, the absence of a panoramic view and the “upside- down” image. Direct visualisation provides an accurate diagnosis. US exams frequently miss pelvic and/or ovarian endometriosis in the early- stages, which bipolar electrosurgery can treat with minimal trauma. When endometriosis was detected, lesions were coagulated using the bipolar probe. We could not be certain that treating these lesions had a beneficial effect on the spontaneous pregnancy rate (i.e. 25.6%). In the absence of biomarkers, it remains unclear whether these lesions would have continued to grow or would have resolved over time. In absence of a direct visualisation of the pelvis minimal endometriotic lesions and adhesions will frequently be missed, therefore, studies in assisted reproductive technology databases must be interpreted very carefully as they can hide undiagnosed pathologies.
